# White matter pathology and disconnection in the frontal lobe in cerebral autosomal dominant arteriopathy with subcortical infarcts and leukoencephalopathy (CADASIL)

**DOI:** 10.1111/nan.12073

**Published:** 2013-07-12

**Authors:** L J L Craggs, Y Yamamoto, M Ihara, R Fenwick, M Burke, A E Oakley, S Roeber, M Duering, H Kretzschmar, R N Kalaria

**Affiliations:** * Institute for Ageing and Health, Newcastle University, Campus for Ageing and VitalityNewcastle upon Tyne, UK; † Department of Stroke and Cerebrovascular Diseases, National Cerebral and Cardiovascular CenterOsaka, Japan; ‡ Institut für Neuropathologie, LMU MünchenMünchen, Germany; § Institute for Stroke and Dementia Research, LMU MünchenMünchen, Germany

**Keywords:** CADASIL, cognitive impairment, disconnection, stroke, vascular dementia, white matter changes

## Abstract

**Background:**

Magnetic resonance imaging indicates diffuse white matter (WM) changes are associated with cognitive impairment in cerebral autosomal dominant arteriopathy with subcortical infarcts and leukoencephalopathy (CADASIL). We examined whether the distribution of axonal abnormalities is related to microvascular pathology in the underlying WM.

**Methods:**

We used *post-mortem* brains from CADASIL subjects and similar age cognitively normal controls to examine WM axonal changes, microvascular pathology, and glial reaction in up to 16 different regions extending rostro-caudally through the cerebrum. Using unbiased stereological methods, we estimated length densities of affected axons immunostained with neurofilament antibody SMI32. Standard immunohistochemistry was used to assess amyloid precursor protein immunoreactivity per WM area. To relate WM changes to microvascular pathology, we also determined the sclerotic index (SI) in WM arterioles.

**Results:**

The degree of WM pathology consistently scored higher across all brain regions in CADASIL subjects (*P* < 0.01) with the WM underlying the primary motor cortex exhibiting the most severe change. SMI32 immunoreactive axons in CADASIL were invariably increased compared with controls (*P* < 0.01), with most prominent axonal abnormalities observed in the frontal WM (*P* < 0.05). The SIs of arterioles in CADASIL were increased by 25–45% throughout the regions assessed, with the highest change in the mid-frontal region (*P* = 0.000).

**Conclusions:**

Our results suggest disruption of either cortico-cortical or subcortical-cortical networks in the WM of the frontal lobe that may explain motor deficits and executive dysfunction in CADASIL. Widespread WM axonal changes arise from differential stenosis and sclerosis of arterioles in the WM of CADASIL subjects, possibly affecting some axons of projection neurones connecting to targets in the subcortical structures.

## Introduction

Cerebral autosomal dominant arteriopathy with subcortical infarcts and leukoencephalopathy (CADASIL) is the most common form of hereditary small vessel disease (SVD), and is linked to mutations in the *NOTCH3* gene 1. The clinical features in CADASIL are characterized by recurrent strokes, migraine with aura, motor deficits, pseudobulbar palsy, mood disturbances and subcortical dementia 1. The profile of cognitive impairment in CADASIL resembles that in sporadic vascular cognitive impairment (VCI), and manifests as deficits in attention, processing speed and executive function, but relatively preserved semantic fluency 2. CADASIL subjects exhibit rather specific spatial distribution of white matter (WM) changes as shown by magnetic resonance imaging (MRI) suggesting disrupted cortical connectivity underlies the cognitive deficits. Abnormalities in normal-appearing WM are not readily demonstrable with conventional MRI, but become visible with diffusion tensor imaging (DTI) or magnetization transfer imaging. However, WM hyperintensities on normal MRI did not correlate with cognitive dysfunction in CADASIL 3. In contrast, DTI was shown to relate to impairment in executive function in SVD as well as CADASIL 4,5. Furthermore, DTI histogram metrics were used to predict disease progression in CADASIL 6,7.

We have previously shown that WM changes are reflected by severe demyelination, and are associated with profound microvascular degeneration and enlarged perivascular spaces in the temporal poles of CADASIL subjects 8. However, the extent of axonal abnormalities has not been described in CADASIL and how this relates to the underlying arteriopathy in the damaged WM is not clear. Here, we performed a systematic pathological examination of the axonal integrity and microvascular changes to provide insights into the vulnerable brain regions of CADASIL subjects prior to death.

## Materials and methods

### Subjects

Table 1 provides the demographic details of the subjects. The mean ages of the CADASIL and control subjects were not different. Available case notes and radiological reports indicated CADASIL subjects showed extensive WM changes consistent with SVD of the brain and met the minimum criteria for cognitive impairment used in our post-stroke survivors study 9. Duration of disease was defined as the time between disease onset at first stroke and death 10. CADASIL diagnosis was confirmed by the presence of *NOTCH3* gene mutations or the presence of granular osmiophilic material (GOM) in arteries within skin biopsies 8. None of the controls had neurological or pathological evidence for cerebrovascular disease or neurodegenerative disorder. We also endured that controls did not have any evidence of cardiovascular disease in life or at autopsy. Brain tissues from CADASIL subjects and controls were collected from four sources. These were the Newcastle Brain Tissue Resource, Newcastle General Hospital; MRC London Brain Bank for Neurodegenerative Diseases; the MRC Sudden Death Brain and Tissue Bank, the University of Edinburgh and Ludwig Maximilians University, Germany. Use of brain tissue was approved by the local research ethics committee of the Newcastle upon Tyne Hospitals NHS Foundation Trust, the Newcastle Brain Tissue Resource (NBTR) committee, and the ethics committees overseeing the Brain Banks at the other respective sites.

**Table 1:** Demographic details of the CADASIL (cerebral autosomal dominant arteriopathy with subcortical infarcts and leukoencephalopathy) subjects and controls

Group (*n*)	Age (years)	Gender	Mutation	Duration (years)	Notable clinical features and risk factors
CAD1	44	F	Arg153Cys	8	Cardiac arrhythmias
CAD2	53	F	Arg133Cys	6	No vascular risk
CAD3	55	M	Arg558Cys	11	Brief history of gout
CAD4	58	M	Arg985Cys	13	No vascular risk
CAD5	59	M	Arg169Cys	12	No vascular risk
CAD6	61	M	Arg169Cys	10	Obesity (55 years –)
CAD7	66	F	D239_D253del	23	No vascular risk, obesity
CAD8	68	F	Arg133Cys	18	Smoking history
CAD9	68	M	Arg153Cys	28	Smoking, prostate tumour
CAD10	52	M	Arg141Cys	15	No vascular risk
CAD11	74	M	Arg141Cys	12	No vascular risk
CADASIL (11)	58.8 ± 7.4	7M/4F	–		–
Controls (10)	65.7 ± 8.1	3M/7F	–		No significant cerebrovascular or neurodegenerative disorder. No pathological diagnosis

The mini-mental state examination (MMSE) scores for the patients ranged from 12 to 21. Mean age of controls was not significantly different from mean age of CADASIL group (*P* > 0.05). CADASIL cases used for SMI32 axonal analysis are designated as CAD1 to CAD9. Cases CAD10 and CAD11 were included in the sclerotic index, APP, GFAP and white matter score analysis.

F, female; M, male.

### Neuropathological examination and quantitative immunohistochemistry

Formalin fixed paraffin embedded coronal blocks from a total of 16 brain regions (Table 2) were examined. These extended along the rostro-caudal axis of the cerebrum, per atlas of Perry and Oakley 11. The blocks were cut serially at either 30 μm thickness for the three-dimensional stereology study, or at 10 μm thickness for routine tinctorial staining and immunohistochemistry as described previously 8. Macroscopic and microscopic pathology was assessed using standardized protocols as described 12,13. Haemotoxylin and eosin (H&E), luxol fast blue (LFB) and cresyl fast violet (CFV) were used as standard stains for describing neuropathological changes and for detection of infarcts, presence of WM rarefaction and extent of arteriopathy. Cerebrovascular lesions including SVD pathology were assessed using a grading system as described previously 13. WM pathology was scored on a scale of 0–3 with 0 = normal, 1 = mild, 2 = moderate and 3 = severe. Vascular scores were not significantly different between the two operators (LC and RNK) with over 90% agreement. The mean of the two sets of scores was used in the final analysis.

**Table 2:** Approximate Brodmann areas (BA) corresponding to underlying white matter regions

Block	Coronal level*	Anatomical area
A	4/5	Pre-frontal superior BA 9/46/32
B	8/9	Mid-frontal superior BA 9/8, cingulate BA 24/32 & deep white matter
C	9/10	Cingulate BA 24/32
G	16/17	Trans/entorhinal BA 27/28, OTG BA36 & dentate fascia
K	24	Parietal lateral BA40/22 & deep white matter
R	4/5	Pre-frontal superior half BA 46/45/47/11/32
S	6/7	Frontal lobe BA 8/9//46/45/47/11/12/32/24
Z	14/15	Motor BA 4, pre-motor BA6, cingulate BA 24 & deep white matter
AA	14/15	Temporal BA 20/21/22
AD	18/19	Motor BA 4, sensory BA 3/1/2/5/40 & cingulate BA23/31 & deep white matter
AE	20	Motor BA 4, cingulate BA 23/31 & deep white matter
AG	20	Hippocampus & temporal BA 27/28/35/36/20/21/22/41/42
AI	24	Parietal medial BA7a, cingulate BA23/31 & deep white matter
AJ	24	Parietal inferior BA 17/30/36/19/37/20/21
AK	26	Parieto-occipital lobes BA 7b/39/40/22/21/20/37/19/18/17/31
L	30	Occipital inferior BA17/18/19

* The table interprets which coronal blocks represent the Brodmann areas according to the Newcastle Brain Map 11.

**Figure 1:**
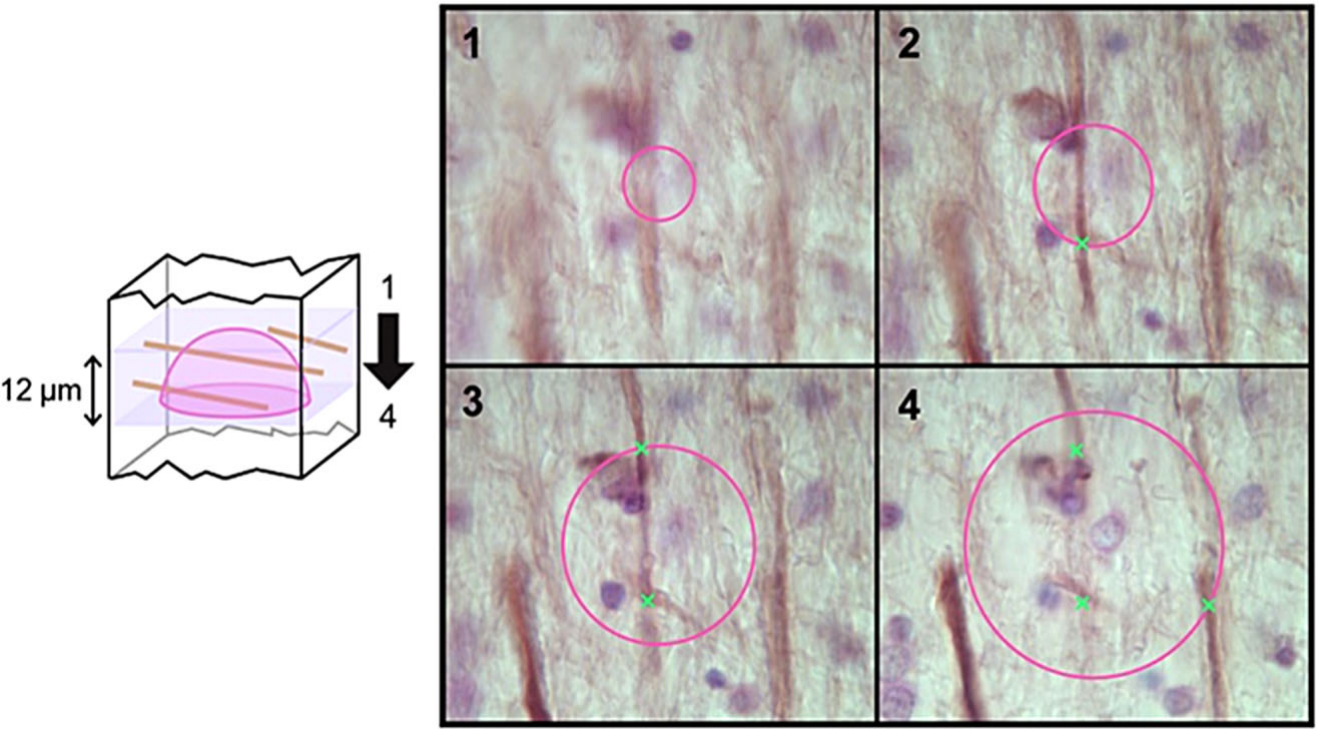
Length density estimation using the space balls method. Hemispherical probes were placed at the centre of tissue thickness. The radius of the probe was set to 12 μm taking the tissue shrinkage and guard volume into consideration. At each sampling point, the sampling depth was scanned through and the intersections between the hemispherical probes (seen as a circle on the screen) and the SMI32 axons in focus (marked with x) were counted. The length density was calculated by dividing the total axonal length by the sampled reference volume.

For SMI32 immunohistochemistry, tissue sections were first immunostained with a mouse anti-nonphosphorylated neurofilament H (anti-SMI32, Covance, CA, USA; 1:500) antibody. The sections were then counterstained with 0.1% CFV to reveal the total cell population (Figure 1). SMI32 is a 200 kDa nonphosphorylated neurofilament protein, expressed in large pyramidal projecting neurones and most abundantly found in the long association pathways connecting the cerebral lobes 14. It is proposed to be a useful indicator of long-term axonal degeneration 15 such that chronically damaged axons exhibit accumulation of SMI32 immunoreactivity 16.

For further immunohistochemical assessments, the following antibodies were used: mouse anti-amyloid precursor protein clone 22C11 (APP, Millipore, Billerica, MA, USA; 1:2000), rabbit anti-glial fibrillary acid protein (GFAP, Dako Cytomation, Glostrup, Denmark; 1:8000) and mouse anti-CD68 (1:200, Dako Cytomation). Unless otherwise specified, sections stained with all other antibodies were counterstained with Haematoxylin. Immunohistochemical staining of different markers was quantified using the per cent area or the region of interest (ROI) method using Image Pro Plus (V.4.0; Media Cybernetics, Silver Spring, MD, USA). For sections stained with GFAP, a grid of 0.5 mm^2^ area was placed over the ROI and the number of cell bodies stained with GFAP was counted from 10 images per ROI.

For additional evidence of axonal damage that complements SMI32 immunoreactivity findings, a subset of sections taken from the regions which scored high and low and indicating large differences between controls and CADASIL cases were immunostained for APP. The degree of accumulated APP was defined by a five point scale as follows: 1 = no staining, 2 = single swollen axons were visible but no punctate deposits, 3 = appearance of punctate deposits within normal appearing WM, 4 = multiple larger areas of punctate deposits and 5 = confluent areas of damaged WM with large areas of multiple APP-stained swollen axons and punctate deposits.

For sclerotic index (SI) analysis and assessment of WM damage, adjacent sections for those used for anti-SMI32 analysis were cut to 10μm thickness and stained with H&E. The SI of microvessels within the WM was essentially determined as described previously 17. We analysed subcortical and deep WM across the four cortical lobes as specified in the results (Table 2). SI values of greater than 0.3 are considered to be a state of mild SVD, and those greater than 0.5 are considered to be severe 18. All arterioles with external diameters between 35 and 350 μm within the region of interest were imaged using a Zeiss Axioplan 2 microscope and Image capture software (Infinity Capture V4.6.0, Lumenera Corporation, Ottawa, Canada).

There were no apparent relationships between the density of immunostaining and length of fixation, or *post-mortem* interval from death to fixation of tissue between the groups. All of the histopathological analyses were performed blind to the operator.

### Unbiased quantification of axonal changes

We used unbiased stereological methods to assess length density of axons revealed by SMI32 immunoreactivity. Immunostained sections were viewed under an Olympus BX51 upright microscope coupled with MBF Bioscience CX9000 camera, and length density of staining was analysed with StereoInvestigator (MBF Bioscience, VT, USA) using a spherical probe (Figure 1). The XYZ motorized stage was controlled by a Modular Automation Controller 6000 (Ludl Electronic Products Ltd, Hawthorne, NY, USA). The sampling grid size was set to 902 × 751 μm to acquire an average of 150–200 counts per ROI in a sample with moderate axonal staining. At each sampling point, the thickness of the tissue was measured using a Heidenhain gauge, and the intersections between the hemispherical probes and the SMI32 axons in focus were counted. The length density, which is defined as total length of axons per unit volume, was calculated by dividing the total axonal length by the sampled reference volume.

### Statistical analysis

Comparisons between markers such as SMI32 length density in CADASIL and controls were made using Kruskal–Wallis and Mann–Whitney *U*-tests incorporated in SPSS version 17.0. WM scores and APP immunoreactivity were further analysed using the independent samples K-Sample Median Test and Kruskal–Wallis *H*-tests in SPSS V19.0. SI was analysed using independent sample *t*-tests and two-way anova followed by Tukey's test *post hoc* in SPSS (IBM, New York, USA, v.19.0). GFAP cell counts were tested for between region effects using anova, and between group effects using *t*-tests for independent samples. Relationships between axonal or pathological markers, and demographic factors, were examined using Spearman's ρ (*rho*) correlation.

## Results

### Regional WM pathology in CADASIL

**Figure 2:**
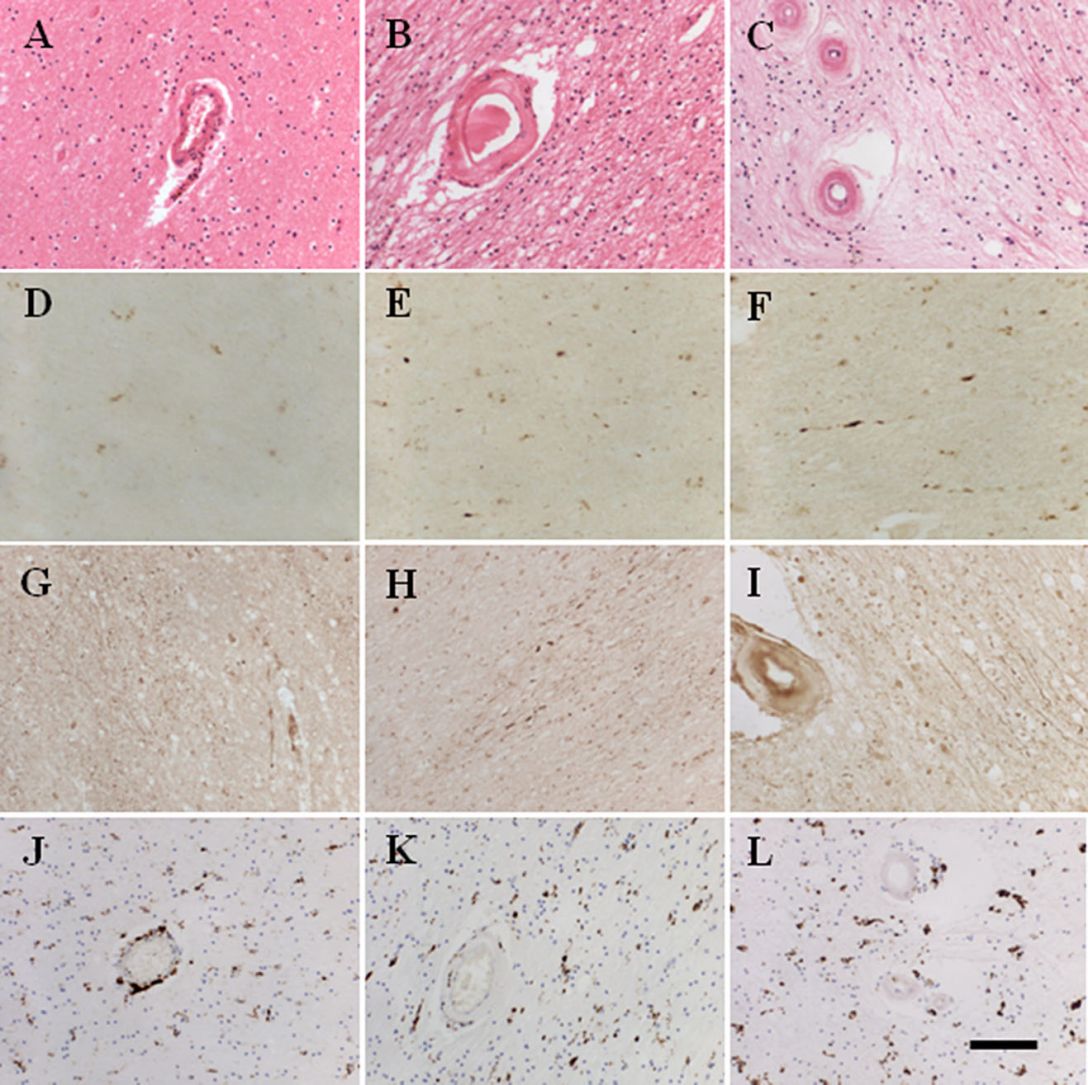
Histopathological features in the frontal and occipital lobes of a 68-year-old CADASIL (cerebral autosomal dominant arteriopathy with subcortical infarcts and leukoencephalopathy) subject. Note more severe white matter (WM) changes and greater SMI32 and APP immunoreactivities in the WM of the frontal lobe (motor) region (**C**, **F**, **I**) compared with the occipital region (**B**, **E**, **H**). A 55-year-old control subject shows mild pathology in the occipital lobe (**A**, **D**, **G**). There were fewer activated microglial cells in controls (**J**) compared with CADASIL subjects (**K** and **L**, occipital and frontal lobes respectively).

Neuropathological examination of the WM in coronal tissue sections from CADASIL subjects revealed the typical microvascular and parenchymal changes. The regional differences in the degree of hyalinosis within arterioles and presence of lacunar infarcts and microinfarcts were readily apparent in CADASIL (Figure 2). Using our previous quantitative assessment methods 13, we observed that the severity of WM pathology consistently scored higher in CADASIL compared with controls (Table 3). There were, however, pathological differences across the brain regions in both CADASIL and control groups (*P* < 0.01), where the WM generally underlying the motor cortex (block Z) scored highly, signifying greater WM damage. However, when WM scores were compared between groups, there were differences between CADASIL and controls in the A, B and K blocks (*P* < 0.05), and trends in changes in the Z and AG blocks (*P* < 0.1), indicating regions specifically affected by CADASIL. Spearman's correlations showed that the degree of WM pathology increased with duration of disease (*rho* 0.308, *P* < 0.05).

**Table 3:** White matter (WM) scores and sclerotic index values across brain regions

Block			A	B	X	Z	AG	K	AK-2	L
Control	WM score	Median	1	1	1	2	1	1	1	1
		Range	0–2	0–2	0–2	1–3	0–2	0–2	0–2	1–2
CADASIL		Median	3	3	2	3	2	3	3	2
		Range	1–3	1–3	2–3	2–3	1–2	2–3	1–3	1–2

Median WM scores as examined using 0–3 scale described in methods, range indicates minimum and maximum scores observed. The block letters in top row correspond to the Brodmann areas (BA) as defined in Table 2, except values for AK-1 and AK-2 were pooled (no significant differences).

CADASIL, cerebral autosomal dominant arteriopathy with subcortical infarcts and leukoencephalopathy.

### Distribution of SMI32-positive axons and APP immunoreactivity

**Figure 3:**
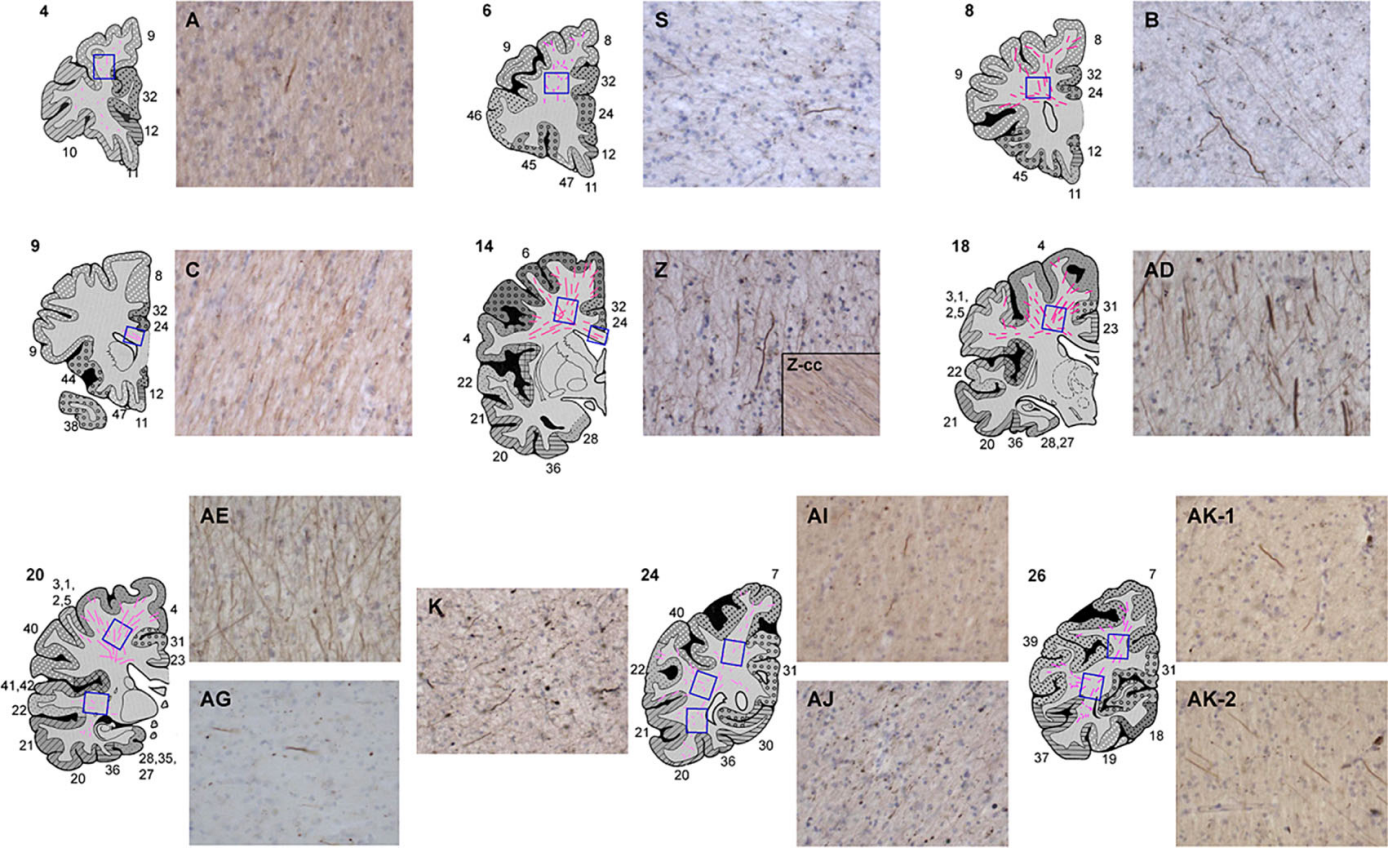
Region of interest (ROI) and representative images of CADASIL (cerebral autosomal dominant arteriopathy with subcortical infarcts and leukoencephalopathy) samples immunostained with SMI32. Extensive staining was found in areas Z, AD and AE. Blue boxes indicate examined areas. Z-cc: corpus callosum area in block Z. Numbers adjacent to cortical slices indicate Brodmann areas.

**Figure 4:**
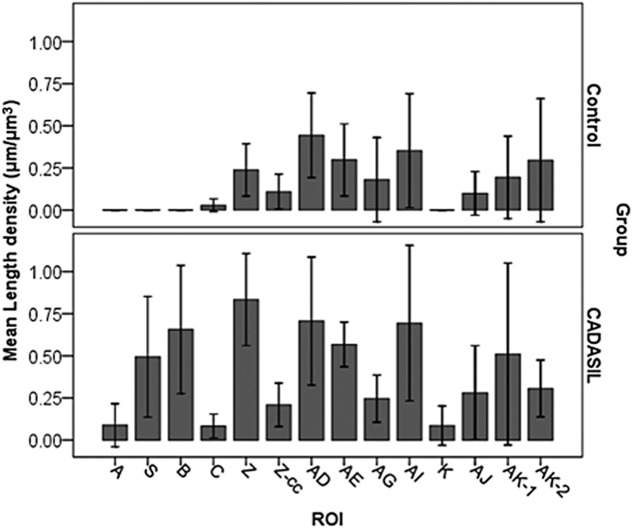
Distribution and density of SMI32-positive axons. In general, cerebral autosomal dominant arteriopathy with subcortical infarcts and leukoencephalopathy (CADASIL) had more axonal staining than controls especially in frontal lobes. The density of abnormal axons correlated with disease duration (see Table 4). Z-cc: corpus callosum area in block Z; ROI: region of interest.

Intense SMI32 immunostaining was observed throughout the WM of CADASIL subjects compared with controls (Figure 3). There was increased frequency of damaged axons particularly in the WM underlying the frontal cortex, BA4 and BA6 regions (Figure 4). In the same regions, microinfarcts and markedly enlarged perivascular spaces were also evident in two of the cases, CAD1 and CAD11. Most surprising was the observation that the highest SMI32 length density was found in the WM at the coronal level of the primary motor cortex (AE and AD), and premotor cortex (Z), in CADASIL subjects, except for one case (CAD11). Thus suggesting that these WM tracts are most vulnerable and undergo intense pathological changes with CADASIL. A large number of stained axons in these areas also showed increased thickening compared with those in other regions. The largest differences between CADASIL and controls were found in the frontal lobe (areas S, B and Z; *P* = 0.029, 0.010 and 0.002 respectively), where controls had no SMI32-positive axons. In addition, surprisingly the SMI32 axonal length density in the temporal lobe (AG) was not as high as in the WM regions close to motor areas, regions AE and AD (Figure 4).

**Table 4:** Correlations between white matter (WM) pathology and markers of axonal damage

Spearman's *rho*		SMI32	*n*	APP	*n*	WM score	*n*	GFAP count/0.5 mm^2^	*n*
SMI32									
APP	NS	23							
WM score	0.517***	40	0.364**	71					
GFAP count/0.5 mm^2^	NS	22	−0.256*	68	NS	69			
Sclerotic index	0.346*	40	0.283*	72	0.543***	125	NS	69	

Values show Spearman's *rho* correlations for number (*n*) values analysed. Statistical significance was designated by following *P* values

*** *P* < 0.001

** *P* < 0.01

* *P* < 0.05.

Abbreviations: SMI32, nonphosphorylated neurofilament H length density; APP, amyloid precursor protein; GFAP count/0.5 mm^2^, glial fibrillary acid protein cell count per 0.5 mm^2^; NS, not significant.

Features of axonal degeneration, such as swollen fragments of axons and ‘retraction bulbs’, were found in areas AG, K and AJ (Figure 3), which likely reduced the intersections with probes and resulted in the lower length density of SMI32 axons in these areas. The severity of the abnormalities differed between some CADASIL cases. CAD11 and CAD12, which had longer duration of disease than CAD1 (Table 1), and correlations between SMI32 length density and disease length in these areas revealed more changes in frontal and parietal lobes (B: *rho* = 0.811, *P* = 0.050; Z: *rho* = 0.987, *P* = 0.001, AI: *rho* = 0.970, *P* = 0.001 and AK: *rho* = 0.978, *P* = 0.022). There were few changes in SMI32 immunoreactivity in the axons within the corpus callosum in CADASIL subjects 19. However, the severity of WM pathology was significantly correlated with SMI32 length density (*rho* 0.517, *P* < 0.001; Table 4).

We further quantified the staining patterns of another marker of axonal change/damage across five brain regions (Figure 2). Overall, axonal APP immunoreactivity scored more highly in CADASIL cases compared with age-matched controls (*P* = 0.014; K-Sample Median Test). However, there were no significant differences in APP staining between the subset of brain regions assessed (*P* = 0.346; Kruskal–Wallis *H*-test) in CADASIL. When comparing between CADASIL and controls regionally, we only observed a trend for APP to score more highly in CADASIL in the WM underlying the motor cortex (*P* = 0.095, K-Sample Median Test). APP reactivity was also correlated with WM pathology (*rho* 0.364, *P* < 0.01; Table 4). Interestingly, APP was also increased with duration of disease (*rho* 0.687 *P* = 0.001).

We also immunostained sections with GFAP and CD68 antibodies to assess whether glial reactions were different in the key regions with high SMI32 reactivity (Figure 2). GFAP- labelled astrocytic cell counts were not found to be different across brain regions either in control brains or in CADASIL (*P* > 0.05; anova). There was also no significant difference in GFAP cell counts between groups in any region of the brain, although there was a trend for GFAP-positive cells to be higher in CADASIL in the WM underlying the motor cortex in block Z (*P* = 0.074). Microglial immunostaining with antibodies to CD68 similarly showed more activated cells in CADASIL (Figure 2) where cells appeared rounder and engorged with material, but there were no quantitative differences between groups or across brain regions (*P* > 0.05, anova).

### Distribution of microvascular pathology

**Figure 5:**
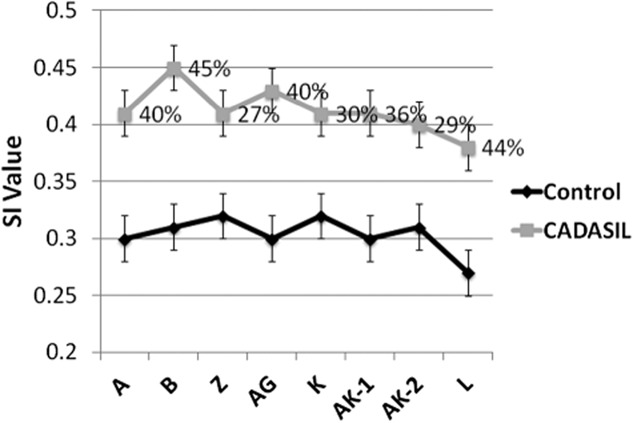
Degree of arteriolosclerosis across white matter (WM) regions in CADASIL (cerebral autosomal dominant arteriopathy with subcortical infarcts and leukoencephalopathy) and normal age-matched control subjects. Points on graph show mean and standard error (bars) of sclerotic index (SI) values in different brain regions. The block letters on *x* axis correspond to the Brodmann areas (BA) as defined in Table 2. The greatest increase in SI was evident in WM underlying mid-frontal lobe (B block).

In an attempt to relate the axonal changes to microvascular pathology, SI values of arterioles were determined in eight selected WM regions from all four lobes of the brain (Figure 5). The mean SI value determined for CADASIL cases was 0.412 (SD 0.05) compared with 0.306 (SD 0.04), in controls, corresponding to an overall increase in CADASIL of 34.5% (*P* < 0.000, Independent samples *T*-test). Two-way anova revealed significant group differences (*P* = 0.000), but only a trend for differences in SI between brain regions (*P* = 0.081), and there was no interaction between groups and brain region (*P* = 0.703). *Post-hoc* tests indicated that again the vessel sclerosis in motor areas in block Z and the temporal lobe in block AG, were different from the occipital lobe in block L (*P* < 0.05, Tukey's test), and showed a trend for differences in the pre-frontal block B (*P* = 0.074).

The largest differences in vessel sclerosis between CADASIL cases compared with controls were 45% and 44.0% in the pre-frontal block B and the occipital lobe in block L respectively, whereas the lowest was in motor areas in block Z and the parieto-occipital region block AK (27% and 29% respectively, Figure 5). When considering the average SIs of arterioles in eight regions across CADASIL brains, our results showed the area with the most significant level of arteriolosclerosis was in the WM underlying the pre-frontal region in block B, with a mean SI of 0.45, followed by the WM in the temporal lobe block AG. All other regions had mean SI values of 0.40–0.41, except for the WM underlying occipital lobe in block L which showed the least degree of arteriolosclerosis in CADASIL brains (mean SI = 0.38), although it was increased by 44% compared with vessels from the same region in controls. Correlational analysis of the whole sample indicated that the SI values were positively associated with the length densities of SMI32 (*rho* 0.346, *P* < 0.05). SI values of blood vessels were also positively correlated with APP reactivity (*rho* 0.283, *P* < 0.05) and WM pathology scores (*rho* 0.543, *P* < 0.001; Table 4).

Further analysis of each of the individual regions showed strong positive association between SMI32 and SI values in the WM underlying the mid-frontal region (*rho* 0.759, *P* = 0.035), and motor areas in block Z (*rho* 0.943, *P* < 0.001). There were no correlations between the SI of arterioles and SMI32 length density in the other regions (data not shown). These results collectively confirmed there is a relationship between axonal abnormalities and microvascular pathologies in the WM of CADASIL patients. However, there are also notable regional differences in the brain where the WM underlying the pre to mid-frontal cortices have greater vulnerability to axon damage.

## Discussion

In this study, our most notable findings were the pathological changes in the WM underlying the motor cortex, where mean length density of SMI32, SI of arterioles, WM scores, and APP immunoreactivity all indicated significant levels of pathological damage in this region. This observation is perhaps not surprising given the indications of motor function abnormalities in CADASIL 6,20. However, the remarkable differential involvement of the brain WM regions even in end-stage CADASIL subjects has not been appreciated from pathological studies 8,10,21,22. We found differential levels of SMI32 staining across regions of both control and CADASIL brains, where specific tracts underlying the primary motor cortex [Brodmann Areas (BA) 4/31] and premotor cortex (BA6) in both controls and CADASIL had the most extensive axonal damage compared with other regions. The WM tracts corresponding to the temporal lobe regions did not reveal profoundly high SMI32 immunoreactivity as would have been expected. This lack of SMI32 reactivity in the temporal lobe further supports the notion that WM hyperintensities observed in the temporal lobe of CADSIL patients are due to enlarged perivascular spaces and are not related to axonal pathogenesis 8.

The most significant increase in axonal abnormalities was detected in the frontal lobe, a region known to be correlated with clinical and neuropsychological profile of SVD-type dementia observed in CADASIL patients 2,23. Our results also indicate that the blood vessels in the WM of the frontal region underlying BAs 9/32 have the highest degree of arteriopathy given the SI analysis. This finding is consistent with also what we reported previously comparing the degrees of microvascular pathology between the basal ganglia and the frontal region 10. While our results advance our previous work on significant degrees of arteriolosclerosis WM of the temporal lobe, frontal and basal ganglia regions 8,10 we also found consistent differential microvascular wall changes across the subcortical WM underlying all four cortical lobes. The correlations between SMI32 length density and SI of vessels showed that there was not complete congruence between the two variables in all regions, and suggest differential disease mechanisms between axon damage and vascular changes. There were robust correlations between SMI32 length density and SI in the WM underlying premotor and primary somatosensory cortices, possibly indicating earlier involvement of these regions in CADASIL disease progression.

As demonstrated with DTI, it is plausible that the SVD type of changes to arterioles in CADASIL cause a chronic hypoxic state within the deep WM resulting from cerebral hypoperfusion 24,25. This likely makes the WM more vulnerable with increased demyelination in older age 26. Given the greater degree of arteriolosclerosis in some WM regions, such a sustained hypoxic state may occur earlier with regional selectivity in CADASIL. However, it is not clear whether axonal damage, as demonstrated by increased SMI32 immunoreactivity, occurs in tandem or secondary to demyelination. We had previously shown that demyelination is associated with accumulation of degraded myelin basic protein 8, which appears to be also differentially distributed in the WM of CADASIL subjects (LC and RNK, unpubl. obs.). Irrespective, our findings support the selective vulnerability of the underlying WM, in tandem with increased stenosis and sclerosis of arterioles in the frontal lobe, as indicated here and implicated by others 22. Our observations are consistent with the fact that the superior longitudinal fasciculus is the most affected WM tract initially rather than the cingulum bundle in CADASIL subjects 24.

The disconnection syndrome is a theory that cognitive dysfunction can be, at least in part, attributed to disrupted associative connections between various brain regions by WM abnormality 27. Tatsch *et al*. 28 have considered disconnections by subcortical lesions as one of the causes of reduced cortical metabolism and cognitive dysfunction in CADASIL. This is consistent with the findings that lacunar infarcts have been associated with cognitive dysfunction in CADASIL but not WM hyperintensities on T2 weighted MRI 3, which we have been shown to reflect fluid accumulation in enlarged perivascular spaces rather than WM loss 8.

A major limitation of our study is that we could not perform robust clinicopathological correlations. We utilized CADASIL brain tissues from several different resources, which inevitably did not allow stringent correlations with clinical features, neuroimaging findings or neuropsychological or cognitive function test scores. Establishing large prospective studies of CADASIL patients followed up to autopsy by collaborating centres would greatly facilitate studies on CADASIL as a model of VCI.

The regional variability in degrees of axonal damage as revealed by SMI32 immunoreactivity suggests that early changes occur in the frontal lobe with a gradual WM disconnection involving the parietal and temporal lobes. It is not yet clear whether these pathological changes precede neuronal atrophy or loss in CADASIL. However, consistent with previous MRI studies on medial temporal lobe atrophy in CADASIL subjects 29, in a parallel study we had found SMI32-positive neuronal densities in CA1 and CA2 regions of the hippocampus tended to be decreased by 30–40% in CADASIL subjects compared with similar age controls (YY *et al*., unpubl. obs.). Collectively, these observations concur with the previously reported vulnerability of the hippocampal pyramidal neurones 30 in vascular dementia in poststroke survivors, indicating there is as a vascular basis for neurodegeneration.

Our results and the previous DTI MRI studies support the hypothesis that cognitive dysfunction in CADASIL is attributable to the disconnection of cortico-cortical or subcortical-cortical networks particularly in the frontal rather than the temporal lobe. These observations have wider implications for cerebral SVD in the elderly.
